# sVCAM‐1 and Hematological Profiles Are Associated With CD4‐Defined Disease Status in HIV Infection

**DOI:** 10.1002/jmv.71037

**Published:** 2026-06-30

**Authors:** Bruno Almeida‐Silva, Claudeir Dias da Silva‐Junior, Victor Vaitkevicius‐Antão, Melayne Rocha Aciole, Milena Márcia da Silva, Líbia Cristina Rocha Vilela Moura, Paulo Sergio Ramos de Araujo, Michelle Christiane da Silva Rabello, Virginia Maria Barros de Lorena

**Affiliations:** ^1^ Department of Tropical Medicine Federal University of Pernambuco Recife Pernambuco Brazil; ^2^ Laboratory of Immunoparasitology, Aggeu Magalhães Institute Oswaldo Cruz Foundation Recife Pernambuco Brazil; ^3^ Translational Research Laboratory Instituto de Medicina Integral Prof. Fernando Figueira (IMIP) Recife Pernambuco Brazil; ^4^ Correia Picanço Hospital Recife Pernambuco Brazil

**Keywords:** biomarkers, HIV, ICAM‐1, selectins, VCAM‐1

## Abstract

Brazil stands out for its progressive public health policies against HIV, including universal access to antiretroviral therapy and a stricter AIDS definition (CD4 < 350 cells/mm^3^) compared to the World Health Organization (WHO) criterion (CD4 ≤ 200 cells/mm^3^). Although CD4 + T‐cell count is the primary marker of immunosuppression, it may not fully reflect the complexity of immune dysfunction during HIV infection. Soluble adhesion molecules, including sVCAM‐1, sICAM‐1, sL‐selectin, sE‐selectin, and sP‐selectin, have emerged as potential biomarkers due to their roles in leukocyte trafficking and endothelial activation. This study primarily consisted of a cross‐sectional analysis, including 127 antiretroviral‐naive people living with HIV (PLHIV) and 40 HIV‐negative controls, complemented by a small longitudinal follow‐up of 33 PLHIV after antiretroviral therapy initiation. PLHIV were categorized into AIDS (CD4 < 350 cells/mm^3^) and NO‐AIDS (CD4 ≥ 350 cells/mm^3^) groups. Molecule concentrations were quantified using Cytometric Bead Array (CBA). Group comparisons and correlations were assessed using Kruskal–Wallis followed by Dunn's multiple comparisons test, Wilcoxon signed‐rank test, and Spearman correlation analysis. Multivariate analyses, including Principal Component Analysis (PCA) and unsupervised K‐means clustering, were performed using R software to explore global patterns in laboratory and adhesion molecule data. sVCAM‐1 levels were significantly lower in the AIDS group compared to both NO‐AIDS individuals and controls (*p* < 0.0001 for both comparisons). ROC curve analysis showed that sVCAM‐1 discriminated AIDS from NO‐AIDS individuals with an AUC of 0.79 (95% CI: 0.7060–0.8769), 67.9% sensitivity, and 86.4% specificity. Although sE‐selectin showed an overall trend toward differences across groups, these differences did not remain statistically significant after correction for multiple comparisons. PCA revealed substantial overlap between groups, with hematological and leukocyte‐related parameters contributing more strongly to multivariate variability than adhesion molecules. Together, these findings suggest that sVCAM‐1 is associated with CD4‐defined disease status and may represent a complementary research biomarker requiring further validation in HIV infection.

## 1 Introduction

Acquired Immunodeficiency Syndrome (AIDS), caused by the Human Immunodeficiency Virus (HIV), is a chronic viral disease that progressively infects and destroys CD4 + T lymphocytes, affecting millions worldwide. The initial infection stage presents high viral load and low CD4 + T cell counts, followed by a temporary CD4+ rebound that never restores pre‐infection levels [1, 2]. This clinical latency period may last months to years until CD4+ counts decline below 200 cells/mm^3^, the World Health Organization (WHO) threshold for AIDS diagnosis. Historically, laboratory diagnosis and monitoring have relied exclusively on this WHO criterion [3].

Brazil has emerged as a global leader in HIV response through comprehensive public policies ensuring universal antiretroviral therapy (ART) access. Notably, Brazil adopts a stricter CD4+ threshold (350 cells/mm^3^) for AIDS classification than the WHO standard (200 cells/mm^3^). This proactive approach reflects Brazil's commitment to mitigating disease progression, reducing transmission, and improving patient outcomes [4, 5].

While CD4+ counts remain a key marker of HIV‐related immunosuppression, the 200 cells/mm^3^ cutoff as a sole diagnostic parameter warrants scrutiny. Individual immune responses vary, with some patients exhibiting elevated cytokine levels or AIDS‐defining conditions despite CD4+ counts above 200 cells/mm^3^, indicating immune compromise before severe CD4+ depletion [6, 7]. These findings suggest CD4+ counts alone may lack sufficient sensitivity as a standalone biomarker. Heterogeneous disease progression further underscores the need for complementary biomarkers to assess immune status and disease severity [8, 9, 10].

Recent research highlights soluble cellular adhesion molecules (CAMs), such as sVCAM‐1, sICAM‐1, sL‐selectin, sE‐selectin, and sP‐selectin, as potential biomarkers in several diseases. In Chagas disease, they have demonstrated strong potential as diagnostic and prognostic biomarkers, particularly in chronic Chagas cardiomyopathy. These molecules have shown effectiveness in distinguishing between clinical stages, including indeterminate, mild, and severe forms, exhibiting high AUC values in ROC analyses [11, 12]. The same CAMs have also been proposed as biomarkers in HIV, where they regulate immune cell migration and inflammation, with emerging evidence linking elevated levels to disease severity and adverse clinical outcomes [8, 13, 14]. However, variations in national ART regimens complicate assessments of their therapeutic modulation.

Key studies demonstrate elevated sVCAM‐1 levels in ART‐naive and ART‐treated PLHIV vs. healthy controls, correlating with disease progression and mortality, particularly in late‐treatment initiators [13, 15]. Graham et al. (2013b) further identified HIV‐induced endothelial activation, suggesting robust virus‐driven inflammation [6].

Selectins also show biomarker potential. Gaddi et al. (2005) reported reduced L‐selectin expression on lymphocytes in HIV‐infected children, implicating its role in immunosuppression [16]. Conversely, Kononchik et al. (2018) found L‐selectin inhibition reduced CD4 + T cell susceptibility to HIV in vitro [14]. While Calza et al. (2009) observed elevated sE‐selectin in untreated HIV patients vs. ART‐treated individuals, Hoffman et al. (2018) detected no significant difference vs. healthy controls, underscoring conflicting evidence on selectins in HIV pathogenesis [13, 17].

Previous studies have demonstrated endothelial activation following HIV acquisition and during antiretroviral therapy, frequently reporting altered levels of soluble adhesion molecules such as sVCAM‐1, sICAM‐1, and selectins. However, most studies compared PLHIV with HIV‐negative controls or evaluated longitudinal changes after infection and ART initiation, with limited emphasis on immunological stratification according to CD4‐defined disease stage. Therefore, this study evaluated serum levels of VCAM‐1, ICAM‐1, L‐selectin, E‐selectin, and P‐selectin in treatment‐naive HIV patients stratified as AIDS and NO‐AIDS, as well as healthy controls. Additionally, we explored the impact of Brazil's first‐line ART regimen (tenofovir/lamivudine/dolutegravir) on these molecules to investigate their potential as complementary biomarkers associated with CD4‐defined disease status.

## 2 Material and Methods

### 2.1 Study Population

This study primarily consisted of a cross‐sectional investigation of treatment‐naive PLHIV stratified according to CD4^+^ T‐cell count. Additionally, a small longitudinal exploratory component was included to evaluate changes in adhesion molecule levels after 2 or 4 months of antiretroviral therapy in independent patient subgroups. This study included 167 individuals of both sexes, aged 18–50 years. Patients with positive serology for syphilis, hepatitis B or C, clinical and/or laboratory diagnosis of tuberculosis, or pregnancy were excluded from the study (Table 1). Peripheral blood samples were collected in dry tubes to obtain serum before and after ART initiation. Samples were transported to the Immunoparasitology Laboratory at Aggeu Magalhães Institute/Oswaldo Cruz Foundation (IAM/FIOCRUZ, Recife, Brazil) for biomarker analysis.

**Table 1:** Characterization of the study population. n.d. – not determined; sex; viral load, CD4 + T cells, CD8 + T cells, and CD4:CD8 are given in the median and first through third quartiles. Differences between groups were evaluated using Kruskal–Wallis followed by Dunn's multiple comparisons test. Data are presented as median and interquartile range (IQR).

	CG	NO‐AIDS	AIDS	*P* (NO‐AIDS vs. AIDS)
*N* = 167	40	64	63	—
Age	24 (23–26)	31.5 (25.5–38)	37 (27–48)	C vs. NO‐AIDS < 0.0001 C vs. AIDS < 0.0001 NO‐AIDS vs. AIDS = 0.01
Sex (%)	M = 12 (30%) F = 28 (70%)	M = 46 (71.87%) F = 18 (28.12%)	M = 37 (58.73%) F = 26 (41.26%)	n.d.
Viral load (copies/mL)	n.d.	25 937 (6071–83 485)	128 816 (39 024–522 937)	0.0003
CD4^+^ T cells (cells/mm^3^)	n.d.	557 (446–705)	132 (51–208)	< 0.0001
CD8^+^ T cells (cells/mm^3^)	n.d.	1212 (796–1552)	680 (511–1136)	< 0.0001
CD4:CD8	n.d.	0.51 (0.32–0.74)	0.17 (0.08–0.26)	< 0.0001

*Note: p* < 0.05 was considered.

We enrolled 127 people living with HIV/AIDS (PLHIV) who received positive diagnoses through two rapid tests conducted at Hospital Correia Picanço (HCP, Recife, Brazil): the ABON HIV 1/2/0 Tri‐line rapid test (Abbott Rapid Diagnostics S.A.) and the Bio‐Manguinhos HIV 1/2 rapid test with Dual Path Platform (DPP) technology (Bio‐Manguinhos/FIOCRUZ), following recommendations from Brazil's Technical Manual for HIV Diagnosis. The study also included 40 healthy controls (negative diagnoses).

The data regarding viral load (VL), CD4 + T lymphocyte count, CD8 + T lymphocyte count, CD4:CD8 ratio, and complete blood count were obtained through patient medical records. The median age of the control group was 24 years (23–26, IQR). One hundred twenty‐seven people living with HIV (PLHIV) formed the groups: AIDS (*N* = 63, CD4 + < 350 cells/mm^3^) with median age of 37 years (27–48, IQR) and NO‐AIDS (*N* = 64, CD4 ≥ 350 cells/mm^3^) with median age of 31 years (25.5–38, IQR).

The AIDS group showed a median of CD4 + T lymphocytes/mm^3^ of 132 (95% CI = 51–208), and the NO‐AIDS group showed a median of CD4 + T lymphocytes/mm^3^ of 557 (95% CI = 446–705). To evaluate the effect of ART on serum levels of these molecules, 16 of the 127 treatment‐naive PLHIV returned after 2 months of treatment and had their samples collected again forming the groups: BT2M (before treatment) and AT2M (after 2 months of treatment) and 17 of the 127 PLHIV returned after 4 months of treatment and formed the groups BT4M (before treatment) and AT4M (after 4 months of treatment).

### 2.2 Quantification of Soluble Adhesion Molecules

Soluble adhesion molecules (sVCAM‐1, sICAM‐1, sL‐selectin, sP‐selectin, and sE‐selectin) were quantified using the BD Cytometric Bead Array (CBA) Human Soluble Protein Flex Set system (BD Biosciences, San Jose, CA, USA), including the Human Soluble VCAM‐1 (CD106) Flex Set (Cat. No. 560427), Human Soluble ICAM‐1 (CD54) Flex Set (Cat. No. 560269), Human Soluble E‐selectin (CD62E) Flex Set (Cat. No. 560419), Human Soluble P‐selectin (CD62P) Flex Set (Cat. No. 560426), and Human Soluble L‐selectin (CD62L) Flex Set (Cat. No. 560420), following the manufacturer's instructions with adaptations to double the yield. Serum samples were analyzed undiluted in a single analytical run to minimize inter‐assay variability and quantified using FCAP Array software v3.0.1 for standard curve generation and concentration estimation. According to the manufacturer's specifications, the assay analytical range was 40–10 000 pg/mL. For samples exceeding the upper limit of quantification, concentrations were extrapolated from the fitted standard curve and interpreted cautiously. Because all samples were analyzed in a single run and as single measurements, intra‐ and inter‐assay variability were not independently assessed. Residual serum was unavailable for repeat dilution measurements of samples exceeding the analytical range.

### 2.3 Statistical Analysis

Patients were primarily classified according to the Brazilian Ministry of Health definition of AIDS (CD4 + T‐cell count < 350 cells/mm^3^), which was adopted as the main grouping criterion because it resulted in a more balanced distribution of participants between groups. To assess the robustness of the findings under the conventional World Health Organization (WHO) definition of AIDS (CD4 + T‐cell count ≤ 200 cells/mm^3^), additional analyses were performed using this alternative cutoff. These results are presented in the Supporting Material (Figure S1).

Continuous variables were represented as median and interquartile range (IQR). The Shapiro–Wilk normality test was used to evaluate data distribution. Comparisons among the control, NO‐AIDS, and AIDS groups were performed using the Kruskal–Wallis test followed by Dunn's multiple comparisons test. Paired comparisons before and after ART initiation were analyzed using the Wilcoxon signed‐rank test. Correlation strength was classified according to Spearman's R values as follows: negligible (0.00–0.29), low (0.30–0.49), moderate (0.50–0.69), high (0.70–0.89), and extremely high (0.90–1.00) [18]. Receiver operating characteristic (ROC) curve analysis was performed to evaluate the discriminatory performance of the studied markers between NO‐AIDS and AIDS groups, including sensitivity, specificity, area under the curve (AUC), and 95% confidence intervals (95% CI). The optimal cutoff value was selected based on the best balance between sensitivity and specificity. AUC values were interpreted as follows: 0.50–0.59, very poor; 0.60–0.69, poor; 0.70–0.79, fair; 0.80–0.89, good; and ≥ 0.90, excellent. P values < 0.05 were considered statistically significant. Data analysis was performed using GraphPad Prism v10.0 (GraphPad Software, San Diego, CA).

### 2.4 Principal Component Analysis (PCA) and K‐Means Clustering

We used R programming language for the PCA and K‐means clustering analysis. The numeric dataset was first standardized using the “scale” function to ensure comparability across variables. Only variables with nonzero variance were retained. The “prcomp” function computed the principal components, and eigenvalues determined the number of components to retain based on the Kaiser criterion (eigenvalues > 1) and the scree plot analysis.

Variable loadings on PC1 were examined to identify key contributors, ranking them by absolute value. Higher loadings indicated a stronger influence on the principal component, with positive and negative values interpreted in relation to biological significance. The PCA results were visualized using biplots and correlation circles. Statistical interpretation considered the clinical relevance of each component, particularly PC1, in differentiating immune and endothelial activation profiles.

To identify patient subgroups based on immune and endothelial activation profiles, K‐means clustering was applied to the PCA results. The optimal number of clusters (K) was determined using the elbow method. The “kmeans” function from the stats package performed the clustering analysis, grouping individuals based on similarity in principal component space. Clusters were visualized using scatter plots of the first two principal components.

## 3 Results

**Figure 1:**
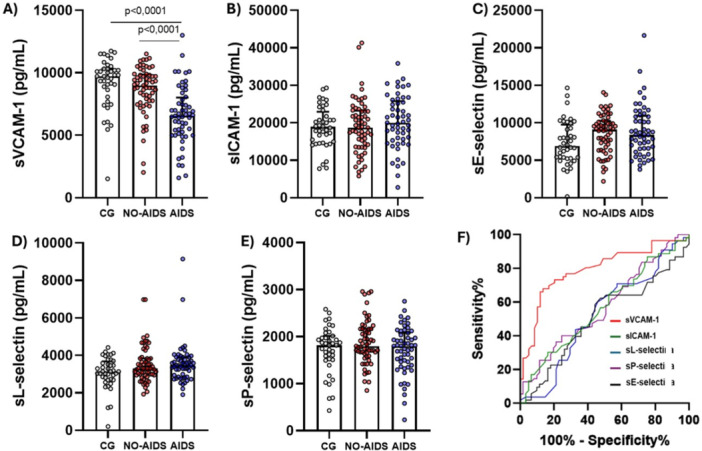
Distribution of soluble adhesion molecule levels and ROC curve analysis according to HIV disease stage. (A) sVCAM‐1, (B) sICAM‐1, (C) sE‐selectin, (D) sL‐selectin, and (E) sP‐selectin serum levels in healthy controls (CG), NO‐AIDS PLHIV (CD4 + ≥ 350 cells/mm^3^), and AIDS PLHIV (CD4 + < 350 cells/mm^3^). Data are presented as scatter plots with median and interquartile range (IQR). Group comparisons were performed using the Kruskal–Wallis test followed by Dunn's multiple comparisons test. (F) Receiver operating characteristic (ROC) curves of soluble adhesion molecules for differentiating AIDS from NO‐AIDS individuals. Sample sizes were CG (*n* = 40), NO‐AIDS (*n* = 64), and AIDS (*n* = 63), except for biomarkers with missing measurements.

We compared sVCAM‐1, sICAM‐1, sL‐selectin, sP‐selectin, and sE‐selectin levels among the control group, AIDS group, and NO‐AIDS group (Figure 1). sVCAM‐1 levels were significantly lower in the AIDS group compared with both the control and NO‐AIDS groups after Dunn's multiple comparisons correction (*p* < 0.0001 for both comparisons), while no significant difference was observed between controls and the NO‐AIDS group. Although the Kruskal–Wallis test suggested overall differences in sE‐selectin levels among groups (*p* = 0.0481), pairwise comparisons did not remain statistically significant after Dunn's correction. No statistically significant differences were observed for sICAM‐1, sL‐selectin, or sP‐selectin. To evaluate whether the choice of CD4 cutoff influenced the observed patterns, the analyses were repeated using the WHO definition of AIDS (CD4 ≤ 200 cells/mm^3^). The overall trends remained consistent with the main analysis, and the corresponding results are presented in the Supporting Material (Figure S1).

To assess the robustness of the main finding, a sensitivity analysis excluding sVCAM‐1 values exceeding the upper analytical range (> 10 000 pg/mL) was performed. The overall pattern of results remained unchanged, with significantly lower sVCAM‐1 levels persisting in the AIDS group compared with both controls (adjusted *p* = 0.0002) and NO‐AIDS individuals (adjusted *p* = 0.0001), while no difference was observed between controls and the NO‐AIDS group.

### 3.1 ROC Curve Analysis

ROC curve analysis was performed to evaluate the discriminatory performance of adhesion molecules between the AIDS and NO‐AIDS groups. Among the evaluated markers, only sVCAM‐1 showed statistically significant discriminatory ability, with an AUC of 0.79 (95% CI: 0.7060–0.8769, *p* < 0.0001). The optimal cutoff value was < 7295 pg/mL, yielding 67.9% sensitivity and 86.4% specificity for distinguishing individuals with AIDS from the NO‐AIDS group (Figure 1F). The remaining adhesion molecules showed poor discriminatory performance, including sICAM‐1 (AUC = 0.56, 95% CI: 0.4586–0.6707, *p* = 0.2351), sL‐selectin (AUC = 0.53, 95% CI: 0.4188–0.6316, *p* = 0.6404), sE‐selectin (AUC = 0.51, 95% CI: 0.4004–0.6175, *p* = 0.8691), and sP‐selectin (AUC = 0.57, 95% CI: 0.4686–0.6775, *p* = 0.1755).

### 3.2 Correlation Among CAMs Concentrations and Laboratory Parameters

In addition to the comparative analysis, we also investigated potential correlations between the studied molecules and clinical markers in the context of HIV. These correlations are shown in Table 2. Among these correlations, sVCAM‐1 showed a weak negative correlation with viral load and lymphocyte count in the NO‐AIDS group and weak positive correlation with CD4 + T lymphocyte levels in the AIDS group. Furthermore, L‐selectin showed a weak negative correlation with lymphocyte count in the NO‐AIDS group, and E‐selectin showed a weak positive correlation with CD8 + T lymphocyte count and viral load in the AIDS group.

**Table 2:** Correlation table among sCAMs and laboratory parameters.

	sVCAM‐1	sICAM‐1	sL‐selectina	sE‐selectina
**AIDS group**				
Viral load (copies/mL)	−0.215	−0.053	−0.121	**0.301** ^a^
CD4^+^ (/mm^3^)	**0.3840** ^**b**^	0.221	0.056	0.105
CD8^+^ (/mm^3^)	0.213	0.134	0.027	**0.381** ^b^
CD4:CD8	0.197	0.058	0.081	−0.135
Leukocytes (×10^3^/mm^3^)	0.115	0.024	−0.035	0.440
Lymphocyte (×10^3^/mm^3^)	0.212	0.150	−0.068	0.284
Neutrophil (×10^3^/mm^3^)	0.057	−0.022	0.031	0.328
Eosinophil (/mm^3^)	−0.075	0.021	−0.033	−0.275
Basophil (/mm^3^)	0.027	−0.113	0.171	0.081
Monocyte (/mm^3^)	0.149	0.130	−0.041	0.210
**NO‐AIDS group**				
Viral load (copies/mL)	**−0.329** ^a^	0.086	0.033	0.019
CD4^+^(/mm^3^)	0.077	−0.06	−0.087	0.047
CD8^+^(/mm^3^)	−0.124	0.108	−0.047	−0.005
CD4:CD8	0.167	−0.13	−0.005	−0.004
Leukocytes (×10^3^/mm^3^)	−0.121	−0.19	−0.113	0.112
Lymphocyte (×10^3^/mm^3^)	**−0.391** ^b^	−0.21	**−0.288** ^a^	0.149
Neutrophil (×10^3^/mm^3^)	0.097	−0.06	0.098	0.003
Eosinophil (/mm^3^)	0.124	−0.22	−0.069	−0.031
Basophil (/mm^3^)	−0.074	−0.24	−0.17	0.227
Monocyte (/mm^3^)	0.240	0.126	0.154	0.082

*Note:* ^a^ *p* < 0.05; ^b^ *p* < 0.005; ^c^ *p* < 0.001. Bold values indicate statistical significance (*p* < 0.05).

### 3.3 Principal Component Analysis (PCA) and Clustering

**Figure 2:**
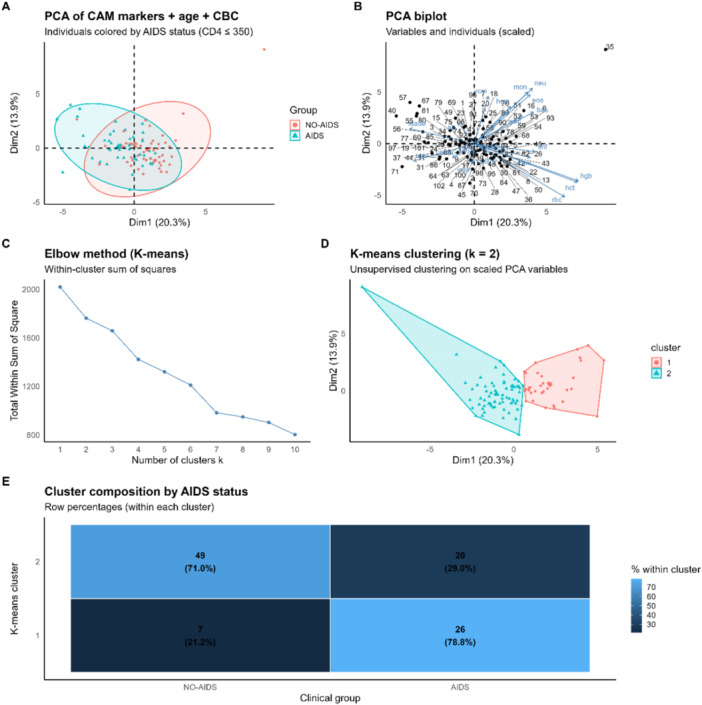
Principal component analysis (PCA) and clustering of CAM markers and laboratory parameters in people living with HIV. (A) PCA score plot showing individuals colored according to clinical classification based on CD4 + T‐cell count (AIDS < 350 cells/mm^3^; NO‐AIDS ≥ 350 cells/mm^3^). Ellipses represent the distribution of each group in the PCA space. (B) PCA biplot displaying both individuals and variable loadings, illustrating the contribution of CAM markers, age, and hematological parameters to the principal components. (C) Elbow method used to estimate the optimal number of clusters based on the within‐cluster sum of squares. (D) K‐means clustering performed on the scaled PCA variables using *k* = 2, illustrating the separation of individuals into two multivariate profiles. (E) Confusion matrix showing the distribution of clinical AIDS status within each cluster, expressed as row percentages.

Principal component analysis (PCA) was performed using CAM markers, age, and complete blood count (CBC) variables to explore global patterns of immune and endothelial activation. The first two principal components (PC1 and PC2) explained 34.2% of the total variance (PC1 = 20.3%; PC2 = 13.9%) (Figure 2A,B).

Visual inspection of the score plot (Figure 2A) demonstrated substantial overlap between the AIDS (CD4 ≤ 350 cells/mm^3^) and NO‐AIDS groups, indicating the absence of a clear multivariate segregation based solely on clinical immunosuppression status. Although the group ellipses showed partial spatial displacement along PC1, a marked interindividual heterogeneity was evident.

To further evaluate multivariate separation, PERMANOVA analysis demonstrated a statistically significant but modest difference between AIDS and NO‐AIDS groups considering CAM markers, age, and hematological variables jointly (*R* ^2^ = 0.067, F = 7.18, *p* = 0.001). PERMDISP analysis showed no significant difference in group dispersion (*p* = 0.1586), suggesting that the observed separation was not primarily driven by unequal dispersion between groups. Similarly, K‐means clusters showed significant multivariate separation (PERMANOVA: *R* ^2^ = 0.127, F = 14.60, *p* = 0.001), with no significant difference in dispersion (PERMDISP *p* = 0.2854). However, silhouette analysis demonstrated limited cluster stability (mean silhouette width = 0.16), reinforcing the exploratory nature of clustering in this study.

The PCA biplot (Figure 2B) revealed that hematological parameters contributed strongly to the separation along PC1, particularly erythrocyte indices (RBC, hemoglobin, and hematocrit), whereas leukocyte subsets (neutrophils, monocytes, and eosinophils) contributed predominantly to PC2. CAM markers showed moderate dispersion across both components, suggesting that endothelial activation does not cluster discretely but rather varies along continuous gradients.

The Elbow method (Figure 2C) suggested limited gain in within‐cluster variance reduction beyond *k* = 2. Accordingly, K‐means clustering was performed with *k* = 2 using the scaled PCA variables (Figure 2D). The resulting clusters were primarily separated along PC1, reflecting differences in combined hematological and CAM‐related profiles rather than strict correspondence with AIDS status. Notably, cluster membership did not perfectly overlap with the CD4‐defined groups, reinforcing the notion that immune and endothelial activation markers distribute along continuous multivariate axes rather than forming sharply distinct biological phenotypes.

The relationship between unsupervised clustering and the clinical classification based on CD4 + T‐cell count (< 350 cells/mm^3^) was further explored using a cluster composition matrix (Figure 2E). Cluster 1 was predominantly composed of individuals classified as AIDS, with 26 out of 33 patients (78.8%) belonging to this group, while only seven individuals (21.2%) were classified as NO‐AIDS. In contrast, Cluster 2 showed the opposite pattern, with a higher proportion of NO‐AIDS individuals (49 of 69 patients; 71.0%) compared to AIDS patients (20 of 69; 29.0%). Although the clusters did not perfectly correspond to the CD4‐based clinical categories, this distribution indicates a partial concordance between the unsupervised multivariate profiles derived from CAM markers, age, and hematological parameters and the conventional immunological classification of disease severity.

### 3.4 Effect of ART on CAMs Levels

**Figure 3:**
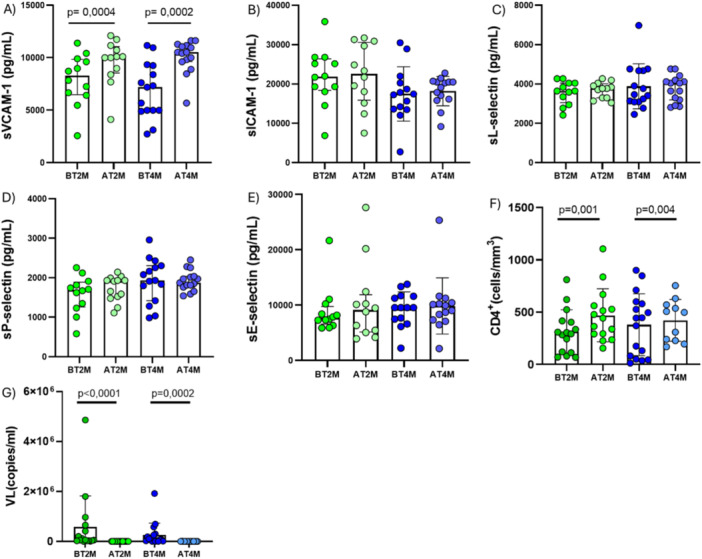
(A) Graph of sVCAM‐1 levels before and after 2 and 4 months of ART treatment; (B) Graph of sICAM‐1 levels before and after 2 and 4 months of ART treatment; (C) Graph of sL‐selectin levels before and after 2 and 4 months of ART treatment; (D) Graph of sP‐selectin levels before and after 2 and 4 months of ART treatment; (E) Graph of sE‐selectin levels before and after 2 and 4 months of ART treatment; (F) CD4 + T‐cell counts before and after 2 and 4 months of treatment; and (G) Viral Load before and after 2 and 4 months of ART treatment.

We compared the CAMs levels between treatment‐naive patients before therapy (BT2M) and the same patients after 2 months of treatment (AT2M), as well as between pretreatment patients (BT4M) and themselves after 4 months of treatment (AT4M). Our results demonstrated significantly higher sVCAM‐1 levels after treatment, with increased concentrations observed in both AT2M and AT4M compared to their respective pretreatment groups (*p* = 0.0004 and *p* = 0.0002, respectively) (Figure 3A). In contrast, no statistically significant differences were observed for sICAM‐1 (Figure 3B), sL‐selectin (Figure 3C), sP‐selectin (Figure 3D), or sE‐selectin (Figure 3E) between the evaluated timepoints.

As expected, antiretroviral therapy was also associated with significant immunological and virological changes. CD4 + T‐cell counts increased after both 2 and 4 months of treatment (Figure 3F), whereas viral load showed a marked reduction after the same treatment intervals (Figure 3G). Together, these findings suggest that the first‐line antiretroviral regimen adopted in Brazil may contribute to early immune recovery and viral suppression, accompanied by increased circulating sVCAM‐1 levels. However, these findings should be interpreted cautiously given the limited longitudinal sample size.

## 4 Discussion

HIV infection continues to have global epidemiological importance, with a growing number of cases, especially in poorer countries. Once infected, these patients are followed on an outpatient basis for the rest of their lives. Currently, CD4 + T lymphocyte count below 200 cells/mm^3^ remains the principal laboratory criterion used to define progression to AIDS. However, studies in the literature indicate immune compromise in patients with counts above 350/mm^3^ [6, 7, 19]. Therefore, in this study, we evaluated the association of the molecules sVCAM‐1, sICAM‐1, sL‐selectin, sP‐selectin, and sE‐selectin with the stages of the disease, based on the cutoff point established by the Brazilian Ministry of Health of 350 cells/mm^3^ for the laboratory definition of AIDS.

Our results showed that sVCAM‐1 levels were significantly reduced in the AIDS group compared to the NO‐AIDS group and the control group. However, we did not observe differences in sICAM‐1 levels among the studied groups, suggesting that sICAM‐1 may have limited utility as a complementary biomarker in HIV infection. Our findings are consistent with those of Calza et al. (2009), who compared serum levels of sICAM‐1 and sVCAM‐1 in untreated PLHIV and healthy controls and found no statistical difference in sICAM‐1 levels [13].

A study by Graham et al. (2013) measured sVCAM‐1 and sICAM‐1 levels in plasma obtained from a cohort of female sex workers before and after HIV infection and found increased levels of both molecules after HIV acquisition, associating this increase with worse prognosis [6]. Similarly, Affi et al. (2022) observed that elevated pre‐ART sVCAM‐1 levels were associated with mortality in HIV‐1 infected adults from West Africa [15]. However, important methodological differences may partly explain the divergence between these findings and our results. Previous studies mainly compared PLHIV with HIV‐negative controls or evaluated disease progression following HIV acquisition, whereas our study primarily compared treatment‐naive PLHIV stratified according to CD4‐defined disease stage (AIDS vs. NO‐AIDS). In addition, Graham et al. included only women and some participants received tuberculosis treatment, while ART exposure, coinfections, and population characteristics varied substantially among studies.

One possible biological hypothesis for the reduction in sVCAM‐1 observed in our AIDS group involves altered regulation of matrix metalloproteinases (MMPs), since soluble VCAM‐1 is generated through enzymatic cleavage of membrane‐bound VCAM‐1 (mVCAM‐1) by metalloproteinases involved in extracellular matrix remodeling [20].

Ghorpade et al. demonstrated that HIV infection may downregulate MMP‐9 expression in monocyte‐derived macrophages despite immune activation, potentially altering proteolytic activity [21, 22]. Additionally, Mastroianni et al. suggested that MMP‐9 activity may be inhibited by increased TIMP‐1 levels in PLHIV [23]. Chronic immune activation in HIV has also been associated with altered MMP/TIMP balance, which may influence endothelial adhesion pathways and the release of soluble adhesion molecules. In this context, one possible hypothesis is that reduced circulating sVCAM‐1 in advanced immunological impairment may reflect impaired cleavage of membrane‐bound VCAM‐1. However, because MMPs and TIMPs were not directly measured in the present study, this interpretation should be considered hypothetical and warrants further investigation.

Our ROC curve analysis showed that sVCAM‐1 significantly differentiated AIDS from NO‐AIDS individuals, with an AUC of 0.79 (95% CI: 0.7060–0.8769). Using the optimal cutoff (< 7295 pg/mL), sVCAM‐1 yielded 67.9% sensitivity and 86.4% specificity, indicating fair discriminatory performance. Although this performance does not support immediate clinical implementation, it suggests that sVCAM‐1 may have potential utility as a complementary research biomarker associated with CD4‐defined disease stage. Additional validation in larger and independent cohorts is necessary before clinical applicability can be considered [24].

Principal component analysis (PCA) reinforced the findings observed in direct comparative analyses, allowing the identification of two axes of biological variability represented by PC1 and PC2. Although these components explained only 34.2% of the total variance and substantial overlap between groups was observed, PCA remained useful for identifying underlying multivariate patterns within the dataset. Together with complementary multivariate analyses, these findings suggest modest but biologically meaningful differences between groups, while also indicating that the observed patterns should be interpreted cautiously and primarily as exploratory.

In datasets containing multiple immunological biomarkers, PCA often explains a limited proportion of total variance in the first components. Nevertheless, this approach remains useful for identifying underlying biological patterns and patient subgroups within complex datasets. Previous studies support this point, Storey et al. (2014) used PCA to identify HIV‐1 antibody response patterns associated with disease progression, even with low variance explained per component. Similarly, Kelly et al. (2020) applied PCA followed by hierarchical clustering to define distinct inflammatory phenotypes among patients with advanced HIV, which were independent predictors of arterial stiffness, a clinically relevant outcome [25, 26].

In the present analysis, the first principal component (PC1) primarily reflected variation in erythrocyte‐related hematological parameters, with strong contributions from red blood cell count, hemoglobin, hematocrit, and platelet levels, together with a moderate contribution from sVCAM‐1. Higher PC1 scores therefore appeared to represent a combined hematological and endothelial profile rather than a direct gradient of immunosuppression. In contrast, PC2 represented an axis associated with leukocyte‐related parameters, including neutrophils, monocytes, eosinophils, basophils, and total white blood cell counts. This second component likely reflects differences in leukocyte activation and inflammatory cell composition. Together, these components indicate that variability in the dataset is largely driven by hematological and leukocyte profiles rather than discrete clustering strictly defined by CD4‐based clinical categories.

The clustering observed in the PCA analysis suggests that individuals classified within the same CD4‐based category may still present heterogeneous immunological and hematological profiles. However, given the modest variance explained and visible overlap between groups, these findings should be interpreted cautiously. Rather than indicating clearly distinct biological subtypes, the observed patterns may reflect subtle differences in inflammatory and hematological profiles that are not fully captured by CD4 + T lymphocyte counts alone. Similar observations have been reported by Kelly et al., who identified inflammatory subgroups among PLHIV associated with clinically relevant outcomes [26].

In addition to PCA visualization, the confusion matrix derived from K‐means clustering provided further insight into the relationship between multivariate profiles and conventional CD4‐based clinical classification. Although clusters did not perfectly overlap with AIDS and NO‐AIDS groups, a clear enrichment pattern was observed, with one cluster containing a higher proportion of AIDS individuals and the other predominantly composed of NO‐AIDS patients. PERMANOVA confirmed statistically significant separation between K‐means clusters (*R* ^2^ = 0.127, F = 14.60, *p* = 0.001), while PERMDISP analysis showed no evidence of unequal cluster dispersion (*p* = 0.2854). However, silhouette analysis demonstrated limited cluster stability (mean silhouette width = 0.16), indicating that cluster separation was modest. Therefore, although the clustering results may reflect biologically relevant variation among patients, these findings should be interpreted within the exploratory scope of the present analysis.

Taken together, these findings suggest that HIV infection may involve broader immunological and endothelial alterations beyond CD4 + T‐cell depletion alone. In this context, multivariate patterns involving hematological parameters and sVCAM‐1 may reflect aspects of chronic inflammatory activation and endothelial dysfunction not fully captured by conventional CD4‐based classification.

The ART effect analysis provides additional support for the hypothesis that reduced sVCAM‐1 levels may be associated with advanced immunological impairment, since patients showed a significant increase in this molecule after 2 and 4 months of treatment, concomitant with viral load reduction and immune recovery. Furthermore, the positive correlation between sVCAM‐1 and CD4 + T lymphocyte levels may indicate that higher sVCAM‐1 levels are associated with a better lymphocyte response in these individuals, since sVCAM‐1 plays a role in cell adhesion and leukocyte recruitment to inflammation sites [27, 28].

Regarding selectins, although the Kruskal–Wallis analysis suggested overall differences in sE‐selectin levels across groups, these differences did not remain statistically significant after correction for multiple comparisons. Therefore, our findings do not support a robust association between circulating sE‐selectin levels and CD4‐defined disease stage in HIV infection. Nevertheless, previous studies have suggested a possible role for E‐selectin in HIV‐associated endothelial activation. Graham et al. (2013) reported increased serum E‐selectin levels following HIV acquisition in a cohort of women, supporting the concept of virus‐associated endothelial activation [29]. In contrast, Hoffman et al. (2018) found no statistically significant differences in sE‐selectin levels between PLHIV and healthy individuals, which is more consistent with our corrected findings [17].

Although our data do not support a significant stage‐specific association for sE‐selectin, endothelial activation remains biologically plausible in HIV infection due to chronic inflammatory signaling. During inflammatory responses, membrane‐bound E‐selectin (mE‐selectin) expression may be induced by pro‐inflammatory cytokines such as TNF‐α and IL1‐β, facilitating leukocyte recruitment and vascular permeability. Its extracellular domain may subsequently be cleaved, generating soluble E‐selectin [30, 31, 32]. Therefore, future studies with larger cohorts may be useful to clarify whether sE‐selectin reflects broader inflammatory activation in HIV infection rather than CD4‐defined disease stage.

Although CD4^+^ T lymphocyte count and viral load measurements remain the gold standards for monitoring HIV infection, biomarkers associated with immunological status may provide complementary information regarding disease heterogeneity and endothelial dysfunction. Our findings indicate that sVCAM‐1 levels are associated with CD4‐defined immunological status and respond to antiretroviral therapy, suggesting potential utility as a complementary research biomarker in HIV infection. However, given the moderate discriminatory performance observed and the analytical requirements of CBA‐based quantification, these findings should be interpreted cautiously and require validation in larger, independent cohorts before any potential clinical application can be considered.

## 5 Conclusion

In conclusion, this study suggests that soluble adhesion molecules are associated with immunological alterations in HIV infection and may reflect aspects of endothelial activation linked to disease status. Among the evaluated molecules, sVCAM‐1 showed a significant association with CD4‐defined immunological status and increased after the initiation of antiretroviral therapy. Multivariate analysis further revealed that hematological and leukocyte‐related parameters contribute to the biological variability observed among patients, highlighting the heterogeneity of HIV infection beyond conventional clinical classifications. Together, these findings support the concept that endothelial biomarkers may provide complementary insights into the immunopathogenesis of HIV infection and contribute to a more comprehensive understanding of disease progression.

### 5.1 Limitations

This study has several limitations that should be acknowledged. First, the cross‐sectional design of the main analysis limits the ability to infer causal relationships between adhesion molecule levels and HIV disease progression. Although the longitudinal analysis demonstrated changes in sVCAM‐1 levels following antiretroviral therapy, this component included a relatively small number of paired samples and should therefore be interpreted as exploratory rather than definitive. Second, the control group was younger than the PLHIV groups, which may have influenced endothelial marker levels. However, the principal sVCAM‐1 finding was observed between AIDS and NO‐AIDS individuals, groups that did not significantly differ in age. In addition, information regarding BMI, smoking status, and other inflammatory comorbidities was not consistently available, precluding adjustment for these potential confounding factors. Third, clinical outcomes such as AIDS‐defining illnesses, disease progression, or mortality were not evaluated, preventing direct assessment of the prognostic clinical value of sVCAM‐1. Finally, although PCA revealed biologically meaningful multivariate patterns, the first principal components explained a modest proportion of total variance, which is common in complex immunological datasets but still limits the interpretation of individual components and supports cautious interpretation of clustering findings.

## Ethics Statement

This study was approved by the Research Ethics Committee on Human Subjects of the Aggeu Magalhães Institute/FIOCRUZ under CAAE number 12152019.4.3001.8807 and received authorization from Correia Picanço Hospital/Pernambuco State Health Department.

## Conflicts of Interest

The authors declare no conflicts of interest.

## Supporting information

Supporting File

## Data Availability

The data that support the findings of this study are available from the corresponding author upon reasonable request.
