# Supplementary material for: sVCAM‐1 and Hematological Profiles Are Associated With CD4‐Defined Disease Status in HIV Infection
Source: J Med Virol. 2026 Jun 30;98(7):e71037. doi: 10.1002/jmv.71037 (PMC13316463; doi:10.1002/jmv.71037)

**Figure S1:** Mean levels of cell adhesion molecules in different groups using the WHO cutoff (200 CD4^+^/mm^3^). A) sVCAM-1, B) sICAM-1, C) sE-selectin , D) sL-selectin, E) sP-selectin.

PL


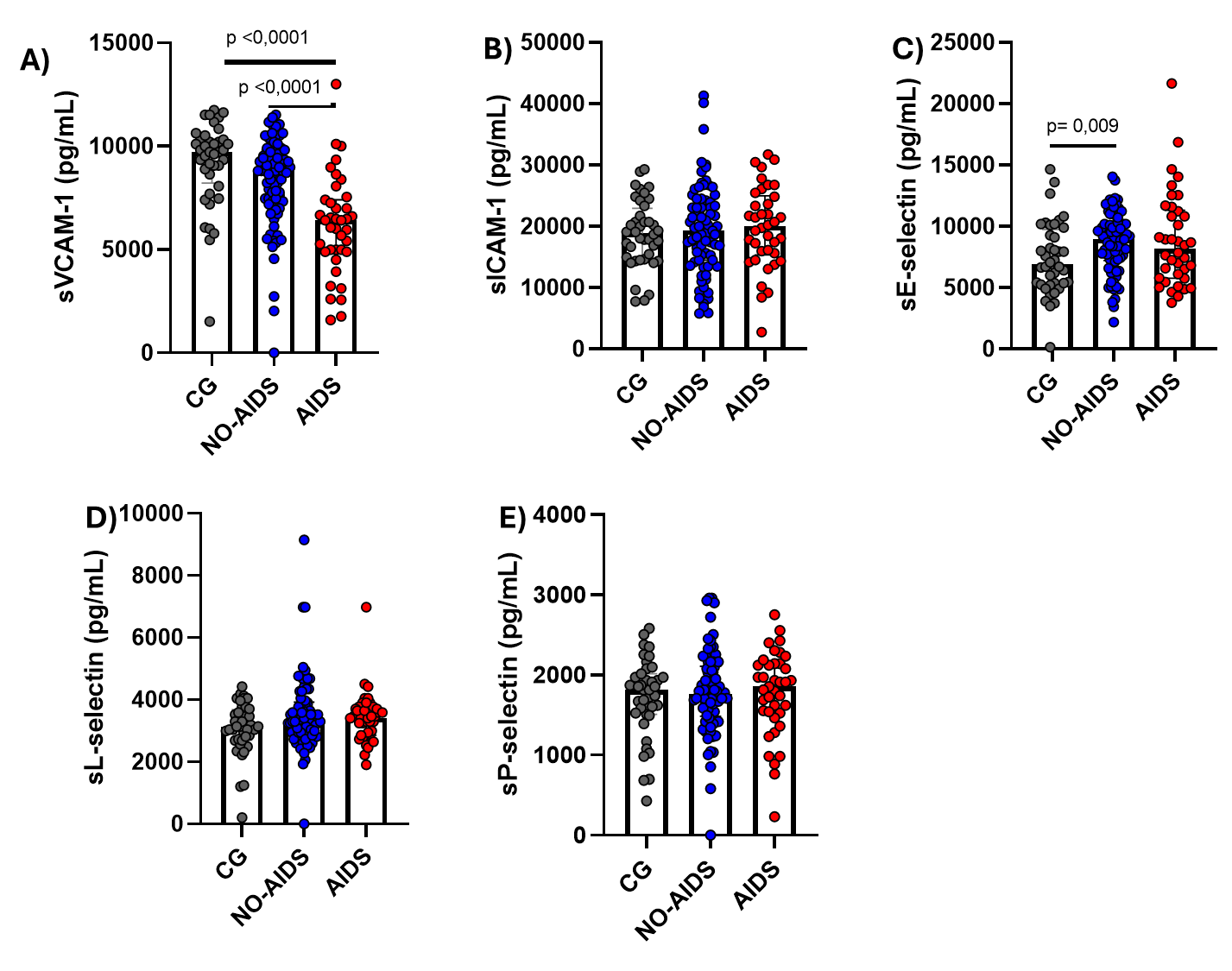

Supplement: Supplementary file 1 — Supporting File [file JMV-98-e71037-s001.docx]
